# Biotin-thiamine responsive basal ganglia disease: a retrospective review of the clinical, radiological and molecular findings of cases in Kuwait with novel variants

**DOI:** 10.1186/s13023-023-02888-y

**Published:** 2023-09-05

**Authors:** Maryam Aburezq, Ahmad Alahmad, Rasha Alsafi, Asma Al-Tawari, Dina Ramadan, Magdy Shafik, Omar Abdelaty, Nawal Makhseed, Reem Elshafie, Mariam Ayed, Abrar Hayat, Fatima Dashti, Dana Marafi, Buthaina Albash, Laila Bastaki, Hind Alsharhan

**Affiliations:** 1 Department of Pediatrics, Farwaniya Hospital, Ministry of Health, Sabah Al-Nasser, Kuwait; 2 Kuwait Medical Genetics Center, Ministry of Health, Sulaibikhat, Kuwait; 3 Department of Pediatrics, Adan Hospital, Ministry of Health, Hadiya, Kuwait; 4 Department of Pediatrics, Al-Sabah Hospital, Ministry of Health, Shuwaikh, Kuwait; 5 Department of Radiology, Farwaniya Hospital, Ministry of Health, Sabah Al-Nasser, Kuwait; 6 Department of Pediatrics, Al-Jahra Hospital, Ministry of Health, Al-Jahra, Kuwait; 7 Department of Neonatology, Maternity Hospital, Ministry of Health, Shuwaikh, Kuwait; 8 Department of Radiology, Adan Hospital, Ministry of Health, Hadiya, Kuwait; 9 Department of Radiology, Ibn Sina Hospital, Ministry of Health, Shuwaikh, Kuwait; 10 Department of Pediatrics, Faculty of Medicine, Health Sciences Centre, Kuwait University, P.O. Box 24923, Safat 13110, Postal Code 90805 Jabriya, Kuwait; 11 Department of Genetic Medicine, Johns Hopkins University School of Medicine, Baltimore, MD USA

**Keywords:** Biotin-thiamine-responsive, Basal ganglia, *SLC19A3*, Neurometabolic, Encephalopathy, Genetic

## Abstract

**Background:**

Biotin-thiamine-responsive basal ganglia disease (BTBGD) is a rare autosomal recessive neurometabolic disorder that is caused by biallelic pathogenic *SLC19A3* variants and is characterized by subacute encephalopathy associated with confusion, convulsions, dysphagia, dysarthria, or other neurological manifestations.

**Methods:**

A retrospective review of the data registry in Kuwait Medical Genetics Center for all cases diagnosed clinically and radiographically and confirmed genetically with BTBGD.

**Results:**

Twenty one cases from 13 different families were diagnosed with BTBGD in Kuwait. Most cases (86%) presented with confusion, dystonia, convulsions, or dysarthria, while three individuals were diagnosed pre-symptomatically during familial targeted genetic screening. Symptoms resolved completely within 2-week of treatment in two-thirds of the symptomatic cases but progressed in six of them to a variety of severe symptoms including severe cogwheel rigidity, dystonia and quadriparesis due to delayed presentation and management. Neuroradiological findings of the symptomatic cases revealed bilateral central changes in the basal ganglia. Two novel homozygous missense *SLC19A3* variants were detected in a Kuwaiti and a Jordanian individuals, in addition to the previously reported Saudi founder homozygous variant, c.1264A > G; p.(Thr422Ala) in the remaining cases. Age of diagnosis ranged from newborn to 32 years, with a median age of 2–3 years. All cases are still alive receiving high doses of biotin and thiamine.

**Conclusion:**

This is the first study reporting the phenotypic and genotypic spectrum of 21 individuals with BTBGD in Kuwait and describing two novel *SLC19A3* variants. BTBGD is a treatable neurometabolic disease that requires early recognition and treatment initiation. This study highlights the importance of performing targeted molecular testing of the founder variant in patients presenting with acute encephalopathy in the region.

**Supplementary Information:**

The online version contains supplementary material available at 10.1186/s13023-023-02888-y.

## Introduction

**Fig. 1:**
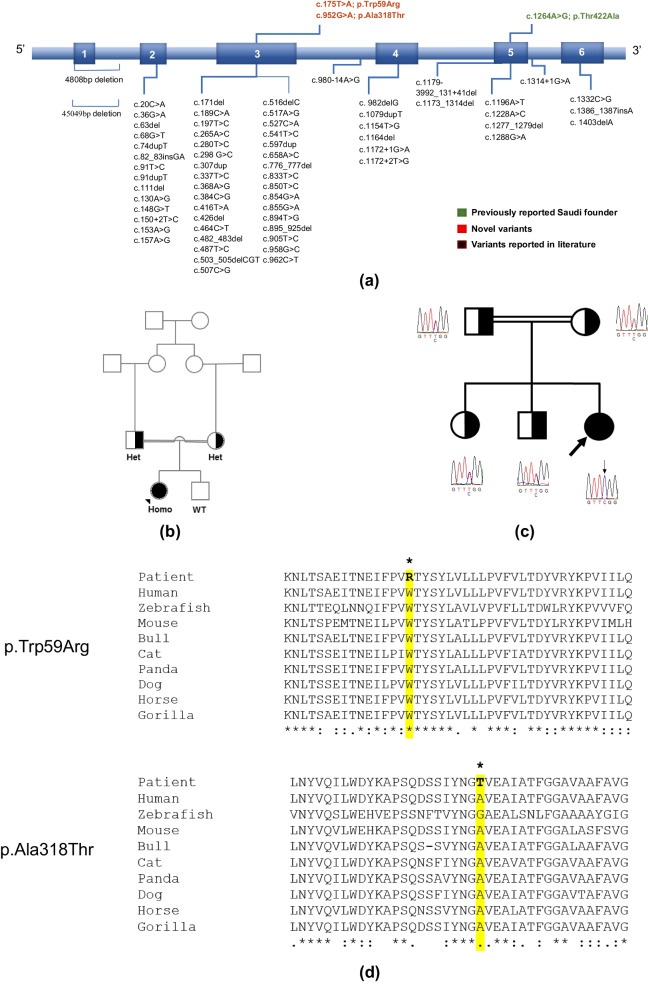
**a** Schematic showing the location of all the *SLC19A3* variants identified in this study and in the literature on the six-exon *SLC19A3* structure (transcript NM 025243.4). The novel variants identified in this study are highlighted in red. The previously reported Saudi founder reported in this study in green. The lower part of the figure represents all the *SLC19A3* variants reported in the literature. **b** Pedigree of Case 9 with segregation of the novel variant c.952G > A;p.(Ala318Thr). **c** Pedigree of Case 15 with segregation of the novel variant c.175T > C;p.(Trp59Arg). **d** Multiple sequence alignment of the two novel variants reported in this study

Biotin-thiamine-responsive basal ganglia disease (BTBGD), also known as thiamine metabolism dysfunction syndrome 2 or thiamine-responsive encephalopathy type 2 (THMD2) (MIM# 607,483), is a rare autosomal recessive neurometabolic disorder [1]. It is a pan-ethnic condition that was first described in 1998 [2] and named biotin responsive basal ganglia disease (BBGD) after the dramatic response of affected cohort to biotin administration [3]. Seven years later, it was found to be caused by biallelic pathogenic variants in *SLC19A3* on chromosome 2q36.3, encoding human thiamine transporter 2 (hTHTR2*)*, affecting thiamine metabolism and transportation through blood brain barrier in the central nerves system [4–6]. It was later discovered that both thiamine and biotin are needed for the treatment, but mainly thiamine [3]. Until now, more than 150 cases with 40 different pathogenic variants in *SLC19A3* were reported worldwide; majority of which were from Saudi Arabia due a Saudi founder variant (Fig. 1a) [7–12].

BTBGD is characterized by subacute encephalopathy that is often triggered by a febrile illness, mild trauma, or other stressors [13]. It manifests with confusion and convulsions, with ataxia, dysarthria, dysphagia, occasional supranuclear facial nerve palsy and/or external ophthalmoplegia. Progressive extrapyramidal signs, including cogwheel rigidity and dystonia, hyperreflexia, ankle clonus and positive Babinski sign, were also reported in some cases, which may also progress to quadriparesis, disability, coma and even death [6, 13, 14].

BTBGD usually manifests in children between three to ten years of age [7, 13]. However, early infantile-onset may occur within the first three months of life with atypical infantile spasm or early infantile Leigh-like syndrome associated with acute encephalopathy, poor feeding, vomiting and severe lactic acidosis, which is usually associated with poor outcome regardless of proper administration of biotin and thiamine supplementation [13]. Moreover, a late-onset disease in the form of adult-onset Wernicke-like encephalopathy with acute status epilepticus and/or the characteristic symptoms of BTBGD could ocurr during the second decade of life [13].

In addition to the clinical manifestations, characteristic neuroradiological findings of bilateral caudate injury with partial or complete involvement of putamen on brain magnetic resonance imaging (MRI) can be suggestive of BTBGD [5]. These findings can be associated with bilateral symmetrical cortical swelling with or without vasogenic edema during acute crisis, while chronic picture may include basal ganglia atrophy and necrosis with diffuse cerebral and to a lesser extent cerebellar involvement [15].

Symptoms typically resolve within few days of administering high doses of biotin and thiamine and may reappear within one month of supplementation discontinuity [2, 8]. We report twenty-one genetically-confirmed BTBGD cases in Kuwait and describe their clinical and radiological findings, along with residual neurological deficits in few individuals with late diagnosis, emphasizing the importance of early diagnosis and timely treatment with high doses of biotin and thiamine. Furthermore, we describe two novel variants in *SLC19A3* in addition to the previously reported Saudi founder variant.

## Materials and methods

### Aim, design and setting of the study

A retrospective chart review to report all individuals diagnosed with BTBGD in Kuwait with their clinical, neuroradiological, and molecular findings obtained from Kuwait Medical Genetic Center (KMGC). Ethical approval for this study was obtained from the ethical committee at the Kuwait Ministry of Health and informed consent was obtained from all participating families.

### Participants characteristics

All individuals with a diagnosis of BTBGD that is confirmed genetically with deleterious biallelic variants in *SLC19A3* were included in our study. Eighteen cases showed clinical and neuroradiological characteristics of the disease prior to confirming the diagnosis genetically, while three individuals were diagnosed pre-symptomatically with family-based targeted genetic screening.

### Clinical findings

All available clinical, laboratory and imaging data, along with the documented neurological and neurodevelopment status were obtained from the medical reports in the KMGC data registry. Medical reports from the corresponding hospital of each individual were also reviewed.

### Neuroimaging and genetic testing

Eighteen individuals had brain MRI performed (18/21, 86%), while it was not performed in the three cases diagnosed pre-symptomatically. All detected twenty-one individuals underwent clinical genetic testing by either targeted variant testing using polymerase chain reaction (PCR) amplification followed by Sanger sequencing (12/21 subjects), full gene sequencing (4/21), through next-generation sequencing technology using Ion AmpliSeq Inborn Errors of Metabolism community panel (ThermoFisher Scientific, Waltham, MA, USA) (2/21 subjects) or clinical exome sequencing (3/21 subjects).

## Results

### Clinical findings

The KMGC registry included 21 affected individuals with BTBGD from 13 different families. All were Kuwaiti nationals, except for a Jordanian and another non-Kuwaiti “Bedoon; i.e. without nationality” individuals. Overview of the detected cases in Kuwait are shown in Tables 1, 2 and Additional file 2 Table S1. Most of these cases but one, were products of consanguineous marriage (20/21; 95%). Both genders were almost equally-affected; 10 males and 11 females. All 21 individuals are currently alive, aging two to 36-year (Additional file 3: Fig. S1), and receiving high doses of oral biotin (5–10 mg/kg/day) and oral thiamine (variable doses reaching up to 40 mg/kg/day, not exceeding 1500 mg/day). Eighteen individuals (86%) presented with subacute encephalopathy, manifested with dystonia, dysarthria, dysphagia, supranuclear facial nerve palsy, confusion and/or convulsions, preceded by a febrile illness or profuse exercise. Most of the cases were diagnosed between the age of two to three-year-old based on genetic testing, while five cases (24%) were diagnosed at a later age of 32, 20, 7, 5 and 4.5-year-old (Additional file 3: Fig. S1). On the other hand, three cases (14%) were diagnosed pre-symptomatically during familial targeted genetic screening and treated immediately, thus they have never been symptomatic. Twelve of the 18 symptomatic individuals (67%) had a complete resolution of their symptoms within two weeks of treatment initiation, while the remaining six individuals (case 1, 2, 5, 7, 14, 15) (33%) had residual neurological manifestations, including severe cogwheel rigidity, dystonia, and dysarthria; two of them (case 5 and 15) (11%) progressed to quadriparesis and disability due to delayed diagnosis and late treatment initiation (Table 2).

**Table 1:** Overview of gender, nationality, molecular and genetic findings of individuals diagnosed with Biotin Thiamine Responsive Basal Ganglia Disease in Kuwait (n = 21)

Case#	Gender	Nationality	Current age (in years)	Symptomatic	Residual deficits	Radiological Findings	*SLC19A3 c.*DNA variant^a^	*SLC19A3* amino acid change	Novel Variant	Consanguinity	Family history	Perinatal history
1	M	K	36	Yes	Yes	+	c.1264A > G (homozygous)	p.Thr422Ala	No	Yes	2 affected cousins (Case 2 &16) Leigh disease Type 1 diabetes mellitus Mitochondrial dysfunction	Gestational HTN on treatment & maternal IDA on iron injections, Child NNJ & RD
2	M	NK	25	Yes	Yes	+	c.1264A > G (homozygous)	p.Thr422Ala	No	Yes	2 affected cousins (case 1 & 16) Cousin with Leigh disease (Case 1)	Gestational HTN on methyldopa
3	F	K	23	Yes	No	+	c.1264A > G (homozygous)	p.Thr422Ala	No	Yes	Affected sibling (Case 4) & niece (Case 19)	Not reported
4	M	K	18	Yes	No	+	c.1264A > G (homozygous)	p.Thr422Ala	No	Yes	Affected sibling (Case 3) & niece (Case 19)	Unremarkable
5	M	K	17	Yes	Yes	+	c.1264A > G (homozygous)	p.Thr422Ala	No	Yes	Unremarkable	Unremarkable
6	F	K	15	Yes	No	+	c.1264A > G (homozygous)	p.Thr422Ala	No	Yes	Affected younger sibling (Case 7) 2 paternal cousins with Leigh’s disease	Unremarkable
7	F	K	15	Yes	Yes	+	c.1264A > G (homozygous	p.Thr422Ala	No	Yes	Suspected elder sibling with clinical and radiological findings of BTBGD but not confirmed genetically	Unremarkable
8	F	K	12	Yes	No	+	c.1264A > G (homozygous)	p.Thr422Ala	No	Yes	Affected sister (Case 6)	Unremarkable
9	F	K	10	Yes	No	+	c.952G > A (homozygous)	p.Ala318Thr	Yes	Yes	Unremarkable	Unremarkable
10	M	K	10	Yes	No	+	c.1264A > G (homozygous)	p.Thr422Ala	No	Yes	Affected sibling (Case 15)	NICU admission for LBW
11	F	K	9	Yes	No	+	c.1264A > G (homozygous)	p.Thr422Ala	No	Yes	2 affected siblings (Case 12&20)	Unremarkable
12	F	K	9	Yes	No	+	c.1264A > G (homozygous)	p.Thr422Ala	No	No	A relative with metabolic disease	Unremarkable
13	M	K	**8**	**No**	N/A	N/A	c.1264A > G (homozygous)	p.Thr422Ala	No	Yes	2 affected siblings (Case 10&20)	Unremarkable
14	M	K	8	Yes	Yes	+	c.1264A > G (homozygous)	p.Thr422Ala	No	Yes	Unremarkable	Unremarkable
15	F	J	7	Yes	Yes	+	c.175T > C (homozygous)	p.Trp59Arg	Yes	Yes	Infant deaths in cousins	PFO, tiny closing PDA
16	M	K	6	No	N/A	N/A	c.1264A > G (homozygous)	p.Thr422Ala	No	Yes	Affected sibling (Case 9) Carrier sibling	ASD
17	F	K	5	Yes	No	+	c.1264A > G (homozygous)	p.Thr422Ala	No	Yes	2 affected cousins (case 1&2)	Unremarkable
18	M	K	4	Yes	No	+	c.1264A > G (homozygous)	p.Thr422Ala	No	Yes	Unremarkable	Unremarkable
19	M	K	3	Yes	No	+	c.1264A > G (homozygous)	p.Thr422Ala	No	Yes	3 early miscarriages Hypothyroidism Absence seizure on Depakene	Maternal hypothyroidism, Maternal aunt with childhood absence seizure on Depakene
20	F	K	2.5	No	No	+	c.1264A > G (homozygous)	p.Thr422Ala	No	Yes	Affected maternal uncle & aunt (Case 3&4)	Unremarkable
21	F	K	2	Yes	N/A	N/A	c.1264A > G (homozygous)	p.Thr422Ala	No	Yes	2 affected siblings (Case 10&12)	Unremarkable

ASD, atrial septal defect; HTN, hypertension; IDA, iron deficiency anemia; IEM; J, Jordanian; K, Kuwaiti; LBW, low birth weight; M, male; NICU, neonatal intensive care unit; NK, Non-Kuwaiti; NNJ, neonatal jaundice; N/A, not applicable; PDA, patent ductus arteriosus; PFO, patent foramen ovale; RD, respiratory distress. *The reference transcript is NM_025243.4

**Table 2:** Overview of the clinical findings, developmental and radiological findings of individuals diagnosed with Biotin Thiamine Responsive Basal Ganglia Disease in Kuwait (n = 21)

Features	Case #	1	2	3	4	5	6	7	8	9	10	11	12*	13	14	15*	16	17	18	19	20*	21	Total (%)
Clinical Features	Unsteadiness/Ataxic gait/ Limping	−	+	+	+	−	+	+	+	+	+	+	+	−	+	−	−	−	+	+	+	−	14/21 (67%)
	Hypertonia/Dystonia/Choreoathetosis/ Contractures	−	+	+	+	+	−	+	−	+	−	+	−	−	+	+	−	−	−	+	+	−	11/21 (52%)
	Irritability/Drowsiness/Lethargy	+	+	+	−	+	−	−	−	−	+	+	+	−	+	+	−	−	−	+	−	−	10/21 (48%)
	Convulsions	+	+	−	+	+	+	+	−	+	−	−	−	−	−	+	−	−	−	−	−	−	8/21 (38%)
	Dysarthria/Nasal speech	−	+	+	+	−	−	+	−	+	−	+	−	−	−	−	−	+	−	−	−	−	7/21 (33%)
	Hypotonia	+	+	−	+	−	−	−	−	−	−	+	−	−	+	+	−	−	−	−	−	−	6/20 (29%)
	Nystagmus/Strabismus/Abnormal gaze	−	−	+	−	−	+	−	−	−	−	−	−	−	−	+	−	−	−	+	+	−	5/20 (24%)
	Tremor	−	+	−	−	−	−	−	−	−	+	−	−	−	+	−	−	+	−	−	−	−	4/21 (19%)
	Drooling	−	+	−	−	+	−	−	−	+	−	−	−	−	−	−	−	−	−	−	+	−	4/21 (19%)
	Scoliosis	−	−	+	−	+	−	+	−	−	−	−	−	−	−	−	−	−	−	−	−	−	3/21 (14%)
	Poor head control	−	+	−	−	+	−	−	−	−	−	−	−	−	−	−	−	−	−	−	−	−	2/21 (10%)
	Chronic constipation	−	−	−	−	+	−	−	−	−	−	−	−	−	−	−	−	−	−	−	−	−	1/21 (5%)
Symptomatic Progression	Residual neurological deficit	+	+	−	−	+	−	+	−	−	−	−	−	−	+	+	−	−	−	−	−	−	6/21 (29%)
	PICU admission	−	−	−	−	−	−	−	−	−	−	−	−	−	+	+	−	−	−	−	−	−	2/21 (10%)
Developmental Status & Somatic Features	GDD/ID	+	−	+	+	+	−	−	−	−	−	−	−	−	−	+	−	−	−	−	−	−	5/21 (24%)
	Dysmorphic features	+	−	−	−	−	−	−	−	−	−	−	−	−	−	−	−	−	−	+	−	−	2/21 (10%)
Radiological Findings	**Basal ganglia changes on brain imaging**	+	+	+	+	+	+	+	+	+	+	+	+	N/A	+	+	N/A	+	+	+	+	N/A	18/18 (100%)

GDD, global developmental delay; ID, intellectual deficiency; N/A, not applicable; PICU, pediatric intensive care unit. *Cases 13, 16, and 21 were diagnosed pre-symptomatically

### Neuroimaging findings

**Fig. 2:**
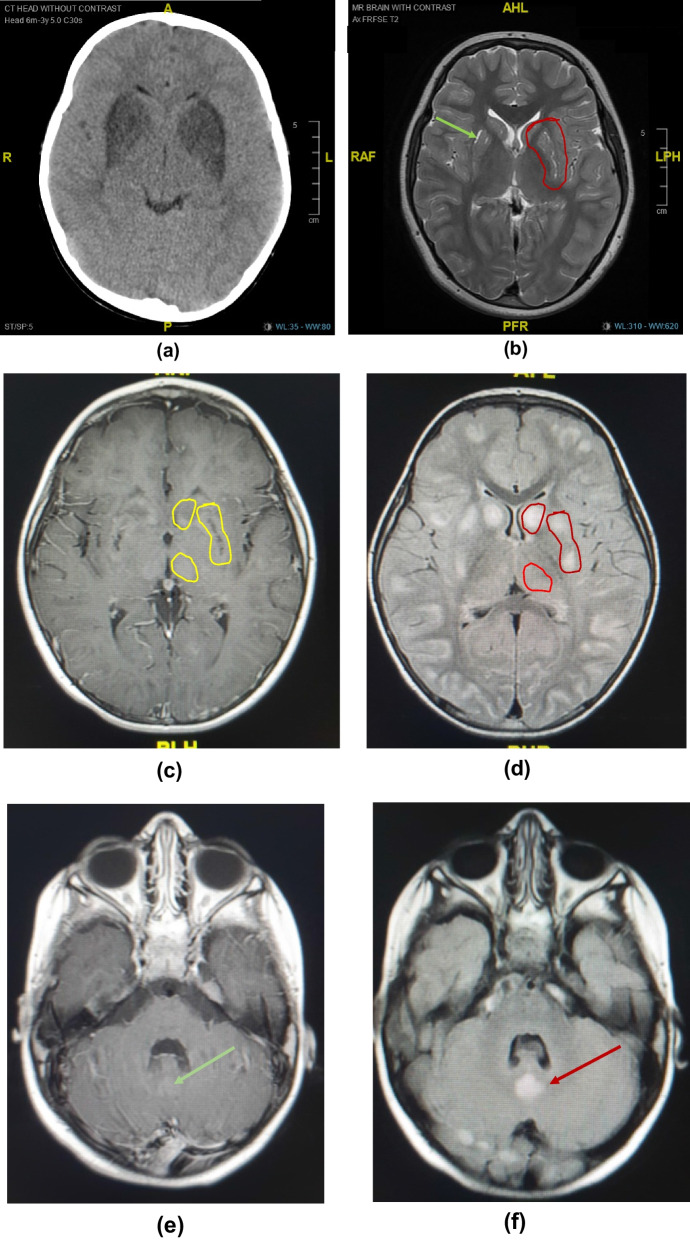

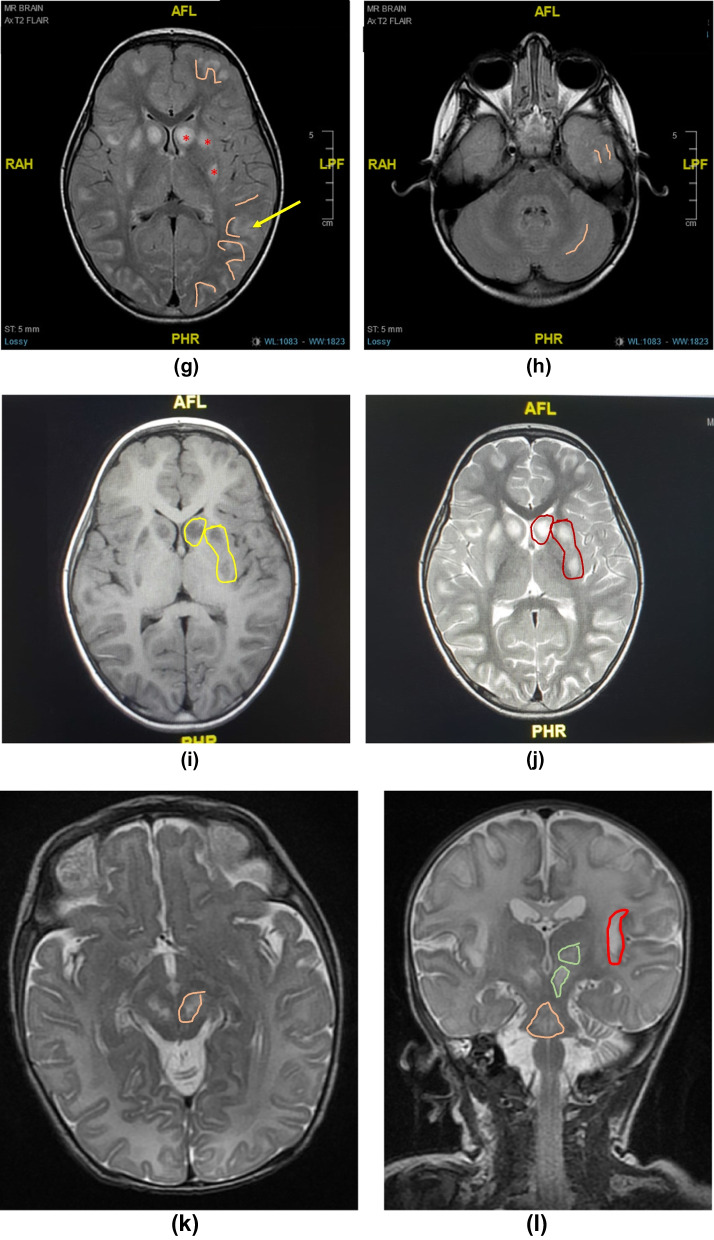

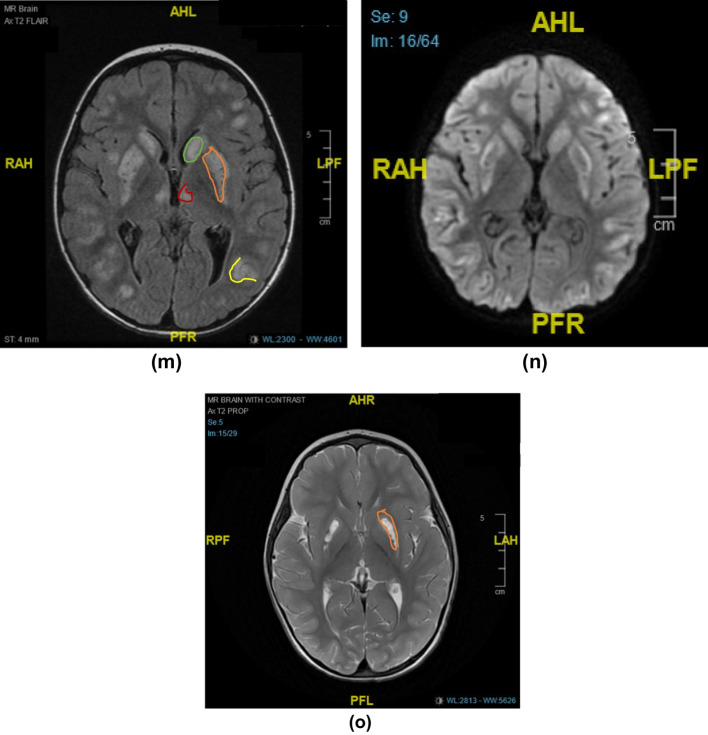
**a**–**o** Radiological Findings of six individuals diagnosed with Biotin Thiamine Responsive Basal Ganglia Disease in Kuwait (Cases 4, 7, 9, 15, 19, 20): **a** Case 4. Plain brain computed tomography (CT) showing bilateral swelling and diffuse hypodensity of putamen. **b** Case 4. Follow up T2-weighted brain magnetic resonance imaging (MRI) revealing bilateral caudate, putamen and external capsule atrophy sparing globus pallidus and sub-insular regions, with multiple T2 hyperintense cystic foci of necrosis/injury. **c** Case 7. T1 post contrast brain MRI showing bilateral T1 faint enhancement in the caudates, peripheral putamen, thalami, and some occipital leptomeningeal regions. **d** Case 7. T2-FLAIR image revealing bright hyperintense lesions again in the basal ganglia and thalami with diffuse bilateral subcortical involvement. **e** Case 7. T1-weighted post contrast image showing faint subtle enhancement of the T2-FLAIR hyperintense lesion in the vermis; (**f**). **g**, **h** Case 9. T2-FLAIR brain MRI showing cortical and subcortical hyperintensities at both cerebral hemispheres and subtle cerebellar changes, as well as caudate and putamen bilaterally. **i** Case 9. T1-weighted brain MRI image correlated with T2-weighted image **j** showing the basal ganglia lesions as T1 hypointense and T2 hyperintense, **k** Case 15. Axial and **l** coronal T2 weighted MR images of the brain showing bilateral symmetric hyperintense signals in the midbrain/cerebral peduncles, as well as the basal ganglia and medial thalami There are multiple cortical/subcortical and bilateral sub-insular T2 hyperintensities. **m** Case 19. T2-FLAIR brain MRI showing multiple scattered hyperintense lesions at the cortical and subcortical cerebral parenchyma, as well as bilateral caudate, putamen and medial thalamic nuclei. **n** Some of these areas showed partial diffusion restriction on DWI. **o** Case 20. T2-weighted brain MRI showing hyperintensity of both putamina, representing atrophy with central necrosis/injury

Most of the 18 symptomatic cases showed similar neuroradiological findings of bilateral hypodensities on the brain computed tomography (CT) and hyperintensities on T2-weighted brain MRI, including the fluid-attenuated inversion recovery sequences (T2-FLAIR), involving mainly the basal ganglia region, along with multiple cerebral cortical and subcortical involvement in some cases, showing edema in acute phases and atrophy and / or injury in chronic phases. Some of the affected areas showed partial diffusion restriction on diffusion weighted brain MRI images (DWI). On T1-weighted MRI, lesions were hypointense in variable degrees, and showed signal void in cases with necrosis/injury. Figure 2i, j of case 9 illustrates most of these findings. Post contrast T1-weighted imaging showed faint enhancement of some lesions compared to bright enhancements in T2-FLAIR images (Fig. 2e, f) in case 7. Brain radiological images of cases 4, 7, 9, 15,19, and 20 are shown in Fig. 2a–o.

### Molecular findings

The diagnosis of the 21 cases was confirmed molecularly; the majority (19/21) had the previously-reported Saudi founder *SLC19A3* variant, c.1264A>G p.(Thr422Ala) (Table 1). Additionally, two novel variants in *SLC19A3* were detected, c. 952G>A; p.(Ala318Thr) in a Kuwaiti individual and c.175T>C; p.(Trp59Arg) in a Jordanian individual, case 9 and case 15 respectively. Both novel variants segregate with the phenotype in the two simplex families (Fig. 1b, c) and are classified as likely pathogenic by ACMG criteria (PP4, PP3, PM2, PS3) based on ACMG Standards and guidelines for the interpretation of sequence variants (Fig. 1d, taken into consideration the clinical and characteristic neuroradiological findings (Table 2). Both novel variants are predicted to be damaging by different computational programs (MetaRNN, BayesDel addA, EVE, Mutation assessor, MutPred, EIGEN, EIGEN PC, FATHMM-MKL, LRT) [16]. All detected variants were inherited from asymptomatic parents that are heterozygous for the pathogenic variant. Figure 1a is a schematic showing the location of all the *SLC19A3* variants identified in this study on the six-exon gene structure, in addition to all *SLC19A3* variants reported in the literature [6, 12, 17–19].

## Discussion

This is the first report from Kuwait describing individuals with BTBGD to bring the total reported individuals to more than 170 since its initial identification in 1998 [2, 7–10]. In this study, we identified 21 new cases from 13 different families with molecularly confirmed diagnosis of BTBGD. The estimated prevalence of BTBGD is approximately 1 in 215,000–1,000,000 individuals with increasing frequencies of detected cases with the expanded knowledge about this disease [20]. Most of our cases were Kuwaiti nationals, except for two individuals. Globally, most of the reported cases were from the Arab population, mainly Saudi due to a homozygous founder variant in *SLC19A3* c.1264A > G; (p.Thr422Ala) [9], with a heterozygous carrier frequency of 1:500 Saudi newborns [21]. This Saudi founder variant was identified as homozygous state in 18 Kuwaiti and one non-Kuwaiti individuals from 11 different families in our study. This is best explained by the high rate of consanguinity among Kuwaiti population reaching over 50%, as the case with the rest of the Middle East Countries as well as the likely high carrier rate of this founder variant in Kuwait [22–25]. The identification of this founder variant in majority of our cases may suggest the possibility of its extension beyond Saudi Arabia and Kuwait to the rest of the Arabian Peninsula. We thus strongly recommend considering molecular screening of this Arab founder variant in all individuals of Arab ancestry presenting with acute encephalopathy. Further cases with about 41 different pathogenic variants from other ethnicities have been reported in the literature, including Canadian, Indian, Mexican, and Western European origin, making it a pan-ethnic disorder [8]. The fact that 90% of the identified BTBGD cases in Kuwait are due to the Saudi founder variant provides a great opportunity for prevention through premarital screening. Such attempt follows the successful model of population-based screening for the founder *HEXA* variants associated with Tay-Sachs disease in the Ashkenazi Jewish population, which has successfully reduced its incidence by 90% [26]. Based on our report, the estimated prevalence of BTBGD in Kuwait with a population of 1,517,076 is about 1 in 77,000 (19/1,517,076) among Kuwaiti, which higher than the reported world prevalence, necessitating its inclusion in future pre-marital screening programs.

We are herein reporting two novel variants in *SLC19A3*, detected in a Kuwaiti and a Jordanian individuals, c. 952G > A; p.(Ala318Thr) and c.175T > C; p.(Trp59Arg) respectively. The Kuwaiti patient with the c. 952G > A variant presented at age 3-year-old with acute limping and dysarthria, with characteristic brain MRI findings of BTBGD (Additional file 2: Table S1). She developed frequent relapses and hospital admissions due to noncompliance and treatment discontinuity (Additional file 1). The Jordanian patent with the c.175T > C variant presented at age of two-month-old with irritability and abnormal movements, early infantile presentation. Brain MRI showed characteristic changes of BTBGD (Additional file 2: Table S1). She developed residual neurological manifestations and was admitted to PICU for relapses due to late diagnosis (Additional file 1). Both novel variants were segregated with the phenotype in the two simplex families (Fig. 1b, c).

There is no clear genotype–phenotype correlation seen among our cases. The correlation between early infantile presentation and c.175T > C; p.(Trp59Arg) variant cannot be assessed on a single case basis and further reported cases are needed. Six of our cases developed residual neurological deficient mainly due to late diagnosis and/or non-compliance to the management of biotin and thiamine (Additional files 1 and 2: Table S1) [8].

The symptomatic cases reported in this study had the classic childhood form of BTBGD except for case 15 that presented as early infantile form. No gender preference as noted among our cohort which is consistent with the literature. Our cases presented with subacute encephalopathy triggered by a febrile illness or profuse exercise, associated with dystonia, dysarthria, dysphagia, supranuclear facial nerve palsy, confusion and/or convulsions, which is in line with the previously reported cases [13, 21, 27]. As previously reported, the median age of diagnosis in our study is 2- to 3-year, except for five cases (24%) were diagnosed late at the age of 32, 20, 7, 5, and 4.5 year-old due to the novelty of this disorder at the time of disease onset (1980s and early 1990s). Similarly, previous studies reported the median age of diagnosis to be around three-year-old [1, 9]. Around two-thirds of the symptomatic cases had a complete resolution within two-week of treatment initiation, while the remaining third had residual rigidity, dystonia, and dysarthria and 11% progressed to quadriparesis and disability due to the late diagnosis and delayed initiation of proper management, as well as non-compliance in some others. Moreover, a literature review revealed that cases that were detected and managed late have progressed to extrapyramidal signs, including cogwheel rigidity, quadriparesis, disability, coma and even to death in some cases [13, 21]. Of note, three cases in our study were diagnosed pre-symptomatically during familial targeted screening as they have at least one affected family member molecularly and clinically diagnosed with BTBGD. They are currently aging 8, 6 and 2-year-old and continue to be asymptomatic, given the early initiation and strict adherence to the high doses of oral biotin and thiamine, highlighting the importance of early detection and management.

The neuroradiological findings in all our symptomatic cases revealed the characteristic bilateral symmetrical central lesions in the basal ganglia, with some cases showing multiple cerebral cortical and subcortical involvement. Similarly, the previously reported key radiological findings of this disease included bilateral symmetrical lesions in the caudate nuclei, putamen and medial thalamus, with variable extension into the brainstem, cerebral cortex and cerebellum [28]. All our reported cases are still alive and receiving biotin and thiamine supplementations. Early administration of high doses of oral biotin (5–10 mg/kg/day) and thiamine (up to 40 mg/kg/day, with a maximum daily dose of 1500 mg) is recommended in suspected cases of BTBGD; in addition to triggers avoidance, and lifelong compliance on taking these supplementations [1, 13].

## Conclusion

BTBGD is a rare but treatable neurometabolic disorder identified in Kuwait and worldwide. It requires a high level of suspicion in any individual presenting with acute encephalopathy associated with abnormal neuroradiological findings in the basal ganglia. Increasing awareness about this treatable condition among clinicians, especially pediatricians, is crucial. A prompt trial of high-dose biotin and thiamine supplementation must be initiated immediately while awaiting genetic confirmation, as timely management significantly impacts the prognosis and prevents progressive neurodegeneration and death.

### Supplementary Information

**Additional file 1**. Review of cases diagnosed with Biotin Thiamine Responsive Basal Ganglia Disease in Kuwait (n = 21).

**Additional file 2: Table S1**. Overview of individuals diagnosed with Biotin Thiamine Responsive Basal Ganglia Disease in Kuwait (n=21). Abbreviations: ASD, atrial septal defect; BG, basal ganglia; Bwt, birth weight; CADASIL, cerebral autosomal dominant arteriopathy with sub-cortical infarcts and leukoencephalopathy; CS, cesarian section; CSF, cerebral spinal fluid; CT, computed tomography; DWI, diffusion-weighted images; FT, full-term; f/u, follow up; FLAIR, fluid-attenuated inversion recovery; GDD, global developmental delay; HTN, hypertension; ID, intellectual disability; IDA, iron deficiency anemia; IEM, inborn errors of metabolism; Kg, kilogram; LBW, low birth weight; MELAS, mitochondrial encephalomyopathy lactic acidosis and stroke-like episodes; MRI, magnetic resonance imaging; MRSA, methicillin-resistant Staphylococcus aureus; MV, mechanical ventilator; NA, not applicable; NGT, nasogastric tube; NICU, neonatal intensive care unit; NNJ, neonatal jaundice; NVD, normal vaginal delivery, PDA, patent ductus arteriosus; PFO, patent foramen ovale; PICU, pediatric intensive care unit; PO, product of; RD, respiratory distress. *The reference transcript is NM_025243.4.

**Additional file 3: Fig. S1**. Age of presentation, age of diagnosis and current age of individuals diagnosed with Biotin Thiamine Responsive Basal Ganglia Disease in Kuwait (n=21).

## Data Availability

The data analyzed during the current study are not publicly available in order to preserve individuals’ privacy. All data included in this study can be shared upon request to the corresponding author (hind.alsharhan@ku.edu.kw).

## Abbreviations

BTBGD: Biotin-thiamine-responsive basal ganglia disease
CT: Computed tomography
hTHTR2: Human thiamine transporter 2
KMGC: Kuwait Medical Genetic Center
MRI: Magnetic resonance imaging
PCR: Polymerase chain reaction
THMD2: Thiamine-responsive encephalopathy type 2
