# Supplementary material for: Biotin-thiamine responsive basal ganglia disease: a retrospective review of the clinical, radiological and molecular findings of cases in Kuwait with novel variants
Source: Orphanet J Rare Dis. 2023 Sep 5;18:271. doi: 10.1186/s13023-023-02888-y (PMC10478457; doi:10.1186/s13023-023-02888-y)
Supplement: Supplementary file 1 — Additional file 1. Review of cases diagnosed with Biotin Thiamine Responsive Basal Ganglia Disease in Kuwait (n = 21). [file 13023_2023_2888_MOESM1_ESM.docx]

**Supplementary Information. Review of cases diagnosed with Biotin Thiamine Responsive Basal Ganglia Disease in Kuwait (n=21).**

In total, 21 cases from 13 different families were clinically, radiologically, and genetically diagnosed with BTBGD in Kuwait. In this review, we are reporting these 21 cases, ordered according to their current ages from the eldest to the youngest individual (1-21).

**Case 1, 2 & 17** are three cousins diagnosed with BTBGD. The eldest reported case of BTBGD in Kuwait is a 36-year-old Kuwaiti male **(Case 1)**, who was diagnosed at age of 32 years. He is a product of full-term pregnancy via spontaneous vaginal delivery (SVD), with a perinatal history remarkable for gestational hypertension and maternal iron deficiency anemia requiring iron injections. After birth, he developed neonatal jaundice and respiratory distress for which he was hospitalized for few days. He is known to have dysmorphic facial features, including hypertelorism, telecanthus, bilateral epicanthic fold synophy, prominent nasal bridge, anteverted nostril, and a high arched palate, in addition to interdigital webbing, long fingers, wide space between the first and second toes and shawl scrotum, but normal male genitalia and normal male karyotyping 46XY. At age of 13-year, he presented with myopathy and recurrent convulsions. Electromyography (EMG) and muscle biopsy revealed a non-specific mitochondrial myopathy, while electroencephalogram (EEG) showed abnormal findings indicative of seizures, which was controlled with valproic acid (Depakene). Five years later, at age of 18-year, he was presented again with generalized mild hypotonia and weakness with slightly diminished deep tendon reflexes. Repeated EMG and muscle biopsy showed normal findings. Both of his brain computerized tomography (CT) and brain magnetic resonance imaging (MRI) showed bilateral signal alteration in lentiform nucleus. EMG was repeated five years later when he was 23-year-old and it showed a picture of myopathy, while brain MRI revealed subacute necrotizing encephalopathy with a suspicion of Leigh Disease. Genetic testing at age of 32-year revealed a homozygous pathogenic *SLC19A3* variant c.1264A>G p.(Thr422Ala) confirming the diagnosis of BTBGD. He has intellectual disability (ID), but able to attend his daily needs with an IQ level of 52 at age of 23 years. Although his school performance was affected, he earned his college degree and started working at age of 30 years. He was born to a consanguineous carrier parents. Family history is remarkable for two affected cousins with BTBGD **(Cases 2 & 17)**, and an older brother with mitochondrial myopathy Leigh disease and his mother was diagnosed with type 1 diabetes mellitus and mitochondrial dysfunction disease. The patient is currently on valproic acid (Depakene), vitamin B6, vitamin B1, clonazepam drops (Rivotril), biotin supplement, coenzyme Q10, and carnitine. He has residual neurological deficit due to delayed diagnosis and treatment initiation.

His cousin is a 25-year-old non-Kuwaiti male (**Case 2)**, who was diagnosed with BTBGD at age of 20 years. He was delivered full-term by cesarian surgery due to prolonged labor with a birth weight of 2.5 kg. His perinatal history was positive to gestational hypertension controlled with Methyldopa (Aldomet). He was completely healthy until the age of 1-year-7-month, when he had acute gastroenteritis episode followed one week later with poor head control, dystonia, generalized hypotonia and weakness, as well as ataxia and falling while walking, progressed to inability to set and walk. His brain MRI revealed bilateral corpus striatum signal alteration with lentiform dystrophic calcification associated with normal brain parenchyma, while his electrocardiogram (ECG), EMG, and EEG tests all were normal. At age of 9-year, he had an upper respiratory tract infection proceeded by his first relapse of BTBGD symptoms, including slurred speech, drooling of saliva, nasal speech, and unsteady gait. Brain CT revealed symmetrical hypodense lesions on BG, while brain MRI showed bilateral hyperintense signals in caudate nucleus, putamen, and frontal gray matter with faint diffused restriction at posterior inferior aspect of lentiform nucleus, in addition to bilateral dystrophic calcification in BG and normal brain parenchyma. Two years later, when he was 11-year-old, he presented again with dysarthria, ataxia, tremors, generalized hypotonia, mild decreased power, brisk deep tendon reflexes, and an upward planter response. Fundus examination was normal, and EEG showed basic activity slower for his age, mainly theta waves with sleeping spindles, otherwise no epileptogenic activity. At age of 18-year, he started to complain of pain with eye movement and brain MRI revealed signs of paranasal sinuses inflammation, including mucosal thickening of maxillary, ethmoidal and right half of sphenoidal sinuses close to the right optic nerve, in addition to hyperintensity in lentiform nucleus, head of caudate and corpus striatum with reduction in size and some atrophic changes, but with no gadolinium enhancing lesions. Two years later, at age of 20 years, he had another relapse with extrapyramidal movements and choreoathetosis involving facial muscle, tongue, extremities, and trunk associated with nasal speech, which improved with symptomatic management. EEG test initially showed burst of generalized slow Delta waves while recording with intermittent photic stimulation and hyperventilation, while repeated EEG after one month came normal. Genetic analysis at age of 20-year confirmed the diagnosis of BTBGD with a homozygous pathogenic *SLC19A3* variant c.1264A>G p.(Thr422Ala). The patient was initially on haloperidol, trihexyphenidyl (Artane), and procyclidine hydrochloride (Kemadrin), which were gradually tapered off after establishing the diagnosis and he was kept on vitamin B1, vitamin B6, vitamin B complex, L-carnitine syrup, and coenzyme Q10 along with the biotin and thiamine supplementation. Three months later, he developed convulsions with mild rare slowing signals over left anterior-mid temporal electrode sites on EEG. Developmentally wise, he had normal developmental milestones and was doing well in high school with normal social interactions. He is a product of first-degree consanguinity. He has a cousin diagnosed with Leigh disease other than the two cousins from his maternal side who were diagnosed with BTBGD (**Cases 1 & 17**).

The third cousin is a 5-year-old Kuwaiti female (**Case 17)*.***  She was diagnosed at age of 1-year-6-month, when she started to develop tremors and limping while walking preceded by chicken pox infection 8 weeks before appearance of symptoms. Genetic analysis revealed a homozygous pathogenic *SLC19A3* variant c.1264A>G p.(Thr422Ala) confirming the diagnosis of BTBGD; thus, she was started on biotin and thiamine supplementations. She was born full-term via SVD to a consanguineous parents with birth weight of 3 kg. Apart of her affected cousins, her parents and two of her siblings are carriers, and she has one healthy sister.

**Cases 3, 4 & 20.** Two Kuwaiti siblings with their niece, who were all diagnosed with BTBGD. The eldest sister (***Case 3***) is a 23-year-old and was diagnosed with BTBGD at age of 2 and a half year. She was born full term via SVD and was totally healthy with normal developmental milestones until the age of two and a half years, when she started to develop progressive weakness, hypertonia, rigidity, dystonia, drooling, upward gaze, ataxic gait, and dysarthria, in addition to eye strabismus and spinal scoliosis. She started to show generalized developmental delay (GDD) associated with intellectual disability (ID) and hyperactivity, for which she studied in a school for special needs till grade of 10 but could not continue due to difficulties in writing with her dystonia. Brain MRI showed bilateral corpus striatum signal alteration with central necrosis at the head of caudate nuclei, restricted diffusion at the outer aspect of lentiform nuclei as well as central dysmorphic calcifications. Molecular diagnosis revealed a homozygous pathogenic genetic variant in *SLC19A3* c.1264A>G p.(Thr422Ala). Her 18-year-old brother ***(Case 4)*** was diagnosed with BTBGD at age of three-year. He was a product of full-term pregnancy via SVD with a birth weight of 2.5 kg. Developmental regression started at age of 2 years and 3 months with slow linearity noticed on reevaluation at age of 6 years, but he had normal cognitive function and social life. He developed two episodes of generalized seizures at age of 3 years, associated with mild hypotonia, slurred speech, ataxia, and dystonic posture during the attacks. Head CT showed bilateral swelling and diffuse hypodensity of putamen. While, brain MRI revealed bilateral caudate, putamen and external capsule atrophy sparing globes pallidus and sub-insular regions, with multiple T2 hyperintense cystic foci of necrosis as well as central dysmorphic calcifications. , and restricted diffusion at the outer aspect of lentiform nuclei in DWI. He was diagnosed with mitochondrial cytomyopathy with dystonic movement disorder and was kept on vitamin B1, vitamin B6, vitamin B complex, vitamin C, L-carnitine syrup, and coenzyme Q10; the later three were stopped and replaced with biotin and thiamine supplementations after establishing the diagnosis of BTBGD. Both affected siblings share the same homozygous pathogenic genetic variant in *SLC19A3* c.1264A>G p.(Thr422Ala) and they are products of first-degree consanguineous carrier parents. They also have an affected niece (***Case 20),*** who is currently 2-year6-month-old. She was born full term via SVD with an unremarkable neonatal history. She was completely healthy with normal developmental milestones until the age of 2-year-3-month when she started to have unsteady gait, bending forward, dystonic movements associated with upward gaze and drooling. She was started on daily 50 mg biotin and 50 mg thiamine supplementation immediately before confirming the diagnosis of BTBGD, due to the reported family history as she had two affected maternal uncles and aunt. MRI findings included hyperintensity of bilateral putamen, representing atrophy with central necrosis. Molecular testing revealed the same genetic variant found in her maternal uncle and aunt.

**Caste 5.** A 17-year-old Kuwaiti male who was diagnosed with BTBGD at age of 5-years He is known to have severe GDD with failure to thrive. No dysmorphic features were reported but he is microcephalic and developed plagiocephaly and scoliosis with time. The patient had recurrent episodes of convulsions and chronic constipation, in addition to hypertonia, lethargy, dystonia, poor head control, drooling and multiple fixed contractures. He is currently wheelchair-bound and unable to set unsupported. The neuroradiological image supported the diagnosis of BTBGD with bilateral symmetrical involvement of the basal ganglia and mild cystic changes at age of 6 years, although it was normal at earlier age. The diagnosis was confirmed genetically with a homozygous pathogenic variant c.1264A>G p.(Thr422Ala) in *SLC19A3*. He was born to contagiousness carrier parents via SVD after completing a full-term pregnancy with birth weight of 3.5 kg and an unremarkable family history. The patient is now on carbamazepine (Tegretol), clonazepam and baclofen, in addition to biotin and thiamine supplementation.

**Cases 6 & 8.** Two Kuwaiti siblings diagnosed with BTBGD. A 15-year-8-month-old female (***Case 6)***, product of full-term SVD with birth weight of 3 kg. She had an unremarkable perinatal and early developmental period until the age of 18-month when she started to have developmental delay and recurrent infections. Later on, she was admitted to hospital for sudden ataxic gait, squint and convulsions. Her CT brain showed multiple hypodense areas in both hemispheres and basal ganglia. Lactate was increased (3.7), but complete blood count (CBC), basic profile, liver profile, ammonia, lactate, thyroid function test (TFT), NeoGen screen, blood amino acids and urine organic acids were normal. Upon obtaining consequent MRI brain, which showed acute diffuse gray matter metabolic disease involving deep gray matter and basal ganglia suggestive of mitochondrial disease. She was started on mitochondrial cocktail after which she markedly improved. She could walk independently, though on wider base and having delayed speech. Until now, she is generally well with appropriate gait and speech, without any neurological deficit, apart of slow learning at school. Brain MRI at age of 2-year revealed bilateral symmetrical swelling of both basal ganglia with abnormal caudate and lentiform nuclei. The affected areas show restricted diffusion on DWI. However, follow up MRI one year and 4 years later were unremarkable. The patient is currently on coenzyme Q10, L-carnitine, vitamin B6, vitamin B1, and B complex supplementation. She is the second child of a healthy consanguineous parents. Her younger sister **(Case 8)** is the third child of these parent, currently 12-year-8month-old, who is a product of a full-term via SVD with a birth weight of 3.5 kg and unremarkable perinatal and early developmental period. At age of 20-month, she was admitted to hospital because of sudden onset of ataxic gait without preceding history of trauma, fits, infection or accidental drugs intake. Mother immediately started her on mitochondrial cocktail, including coenzyme Q10, L-carnitine, vitamin B6, vitamin B1, and B complex supplementation, as her older sister with similar onset of disease has markedly improved on that treatment. She was first time seen at pediatric neurology clinic at 2-year-2-month being already on mitochondrial cocktail treatment and having normal neurological findings. Currently, she is generally well with appropriate gait and speech. Her CBC, basic profile, liver profile, ammonia, lactate, TFT, GeoGen screen, blood amino acids and urine organic acids were normal. Her brain MRI was initially normal, then at age of 6-year it progressed to develop bilateral putamen mild cystic changes. Advanced genetic testing of both siblings revealed 1264A>G p.(Thr422Ala) variant in *SLC19A3*. Regarding their family history, they have two paternal cousins with history of Leigh disease.

**Case 7.** A 15-year-old Kuwaiti girl with a diagnosis of BTBGD since age of 2 and a half year, when she developed fever and diarrhea followed by dysarthria and ataxia that progressed to weakness and inability to walk. Laboratory test was positive to increased serum and CSF lactate level. CT scan imaging showed bilateral near symmetrical hypodensities in both basal ganglia and temporooccipital lobes. T1-weighted brain MRI revealed subtle hypointensities, and post contrast revealed faint enhancement in the caudate, peripheral putamen, thalami and some occipital leptomeningeal regions. While T2-weighted confirmed bright hyperintense lesions again in the basal ganglia and thalami with conspicuous subcortical involvement. She had frequent recurrent episodes of neurodevelopmental abnormalities characterized by convulsions, ataxia and progressive dystonia. She progressed to have dystonic gait with diminished arm swing and dystonic posture, along with mild scoliosis as well as having a positive Babinski sign. Brain MRI showed a persistent diffuse basal ganglion abnormality. Multivocal spectroscopy also revealed a lactate doublet peak in left thalamus. The diagnosis of BTBGD was confirmed at age of 7 years with 1264A>G p.(Thr422Ala) variant in *SLC19A3.* This case’s plan was to continue on lifelong treatment with biotin and thiamine as well as Keppra, and to avoid any metabolic stress, treating fever with prompt appropriate treatment of infections, and providing good nutrition and hydration. However, poor compliance of medication intake with improper dosing provided by the caregiver were reported during her follow up, which could be the reason of her recurrent relapses. She was born full-term via SVD with no perinatal and postnatal complications and normal developmental history. She is a product of a first degree consanguineous parents. Her 29-year-old sister had similar clinical presentation and radiological findings with a suspension of a similar genetic mutation of BTBGD as well; however, no further investigations were conducted. The remaining of her three brothers are healthy and currently aging 31, 27 and 22 years old.

**Case 9.** A 10-year-old Kuwaiti girl, diagnosed with BTBGD at age 3-year-old, when she developed limping and dysarthria for one week. Brain CT showed multiple bilateral hypodensities in temporoparietal and occipital lobes as well as basal ganglia-thalamus complexes, while brain MRI revealed cortical and subcortical hyperintensities at both cerebral hemispheres and subtle cerebellar changes, as well as caudate and putamen bilaterally. Molecular testing confirmed the diagnosis of BTBGD with novel homozygous variant in *SLC19A3*, c.952G>A p.(Ala318Thr). She was born full-term via SVD with normal developmental history. She is a product of a consanguineous parents. She was admitted twice to the hospital for dystonia, convulsions, drooling and inability to walk due to noncompliance and treatment discontinuity by her caregiver.

**Cases 10 & 16.** Two Kuwaiti brothers with a confirmed diagnosis of BTBGD. The eldest brother **(Case 10)** is 10-year-old and was diagnosed at age of 4-year-6-month, when he developed unsteady gait and tremors. Both brain CT and MRI revealed symmetrical bilateral basal ganglia involvement, hypodensity in CT and hyper intensity in MRI, affecting the lentiform nuclei as well as the medial portion of the thalamic nuclei. He had another relapse of acute drowsiness and unsteady gait two years later, at age of 6-year-6-month; otherwise, his symptoms were well controlled with the received biotin and thiamine supplementation. He was born preterm (29-weeks gestation) by SVD with history of neonatal intensive care unit admission for low birth weight of 800 grams. He was reported to have a normal developmental milestones. His younger 6-year-old brother **(Case 16)** was diagnosed pre-symptomatically at age of 2-year-6-month with genetic screening test and he was started on biotin and thiamine supplementation immediately. He is a product of full-term pregnancy, born via SVD with birth weight of 1 kg. He had a history of atrial septal defect which was resolved spontaneously, otherwise he was completely healthy with normal developmental milestones and normal cognitive function. Molecular analysis for both siblings revealed homozygous pathogenic c.1264A>G p.(Thr422Ala) variant in *SLC19A*. They are products of contagiousness carrier parents, and they have another healthy brother.

**Cases 11, 13 & 21.** Three other Kuwaiti siblings with a homozygous pathogenic *SLC19A3* variant c.1264A>G p.(Thr422Ala) confirming the diagnosis of BTBGD. The eldest sibling is a 9-year-old female (**Case 11)**, a product of full-term pregnancy who was born by SVD with birth weight of 3.5 kg. She had normal developmental milestones. She presented with wide base unsteady gait, hypotonia, hyperreflexia, dystonic movements, and dysarthria at age of 2-year. Brain CT showed bilateral symmetrical basal ganglia hypodensities involving the caudate and lentiform nuclei; and brain MRI revealed bilateral symmetrical hyperintensity with diffused restriction and swelling in caudate nuclei and putamen, sparing the globus pallidus. BTBGD was suspected so she was started on biotin and thiamine supplementation immediately before the genetic confirmation. She started to walk, and her symptoms were resolved 8 days after initiation of treatment. Her 8-year-old brother **(Case 13)** was diagnosed at age of one-year while screening the family after diagnosing the eldest sister. He was asymptomatic and never developed any symptom due to early initiation of treatment. No brain imaging was performed. He was born full-term via SVD with macrosomia (>4kg). He is developmentally normal with growth parameters around the 97^th^ centile. Their youngest sister **(Case 21),** is currently 2-year-old, born full-term via SVD as well with birth weight of 3.5 kg. She underwent genetic testing screening for BTBGD after birth and was found to be affected. She was started on biotin and thiamine supplementation pre-symptomatically. She has normal developmental milestone and has never developed any neurological symptom. No brain imaging was performed. The three affected siblings are products of consanguineous carrier parents.

**Case 12.** This is a 9-year-old Kuwaiti female, who was diagnosed with BTBGD since age of 3-year when she developed unsteady gait with falling while walking which resolved after initiation of biotin and thiamine supplementation. Molecular testing revealed a homozygous pathogenic c.1264A>G p.(Thr422Ala) variant in *SLC19A3.* She was born full-term via SVD with birth weight of 3.1 kg. She has normal developmental millstones and cognition. This patient is the first child of her non-consanguineous carrier parents. She has two normal younger brothers and one carrier sister, and a family history of one relative diagnosed with BTBGD.

**Case 14*.*** An 8-year-old Kuwaiti male, who had his first symptomatic presentation at age of 2-year and was diagnosed with BTBGD at age of 3-year. He was born full term via SVD with birth weight of 2.9 kg. He has been growing and developing adequately with good cognition and social interactions. He was admitted with enterovirus, parvovirus, and adenovirus infections at age of 2-year, after which he started to have unsteady gait that slowly progressed to inability to walk or stand without support. His symptoms were associated with hypotonia and weakness in lower limbs and tremors in upper limbs as well as irritability, for which he was admitted to pediatric intensive care unit (PICU). Brain CT revealed hypodense areas in the basal ganglia, while brain MRI showed symmetrical diffusely swollen lentiform and caudate nuclei with lentiform fork sign. A provisional initial diagnosis of autoimmune encephalitis was given with a suspicion of BTBGD, and he was treated with a course of cefotaxime (Claforan), acyclovir, intravenous immunoglobulins, oseltamivir (Tamiflu), biotin, thiamine, and methylprednisolone followed by oral prednisolone. His symptoms were resolved gradually until he was able to walk without support and he was discharged on biotin, thiamine, ranitidine (Zantac) and prednisolone with genetic follow up to rule out BTBGD. At age of 3-year-6-month, he presented again with recurrent choreoathetoid movements. Molecular testing confirmed the diagnosis of BTBGD with a homozygous pathogenic *SLC19A3* variant, c.1264A>G p.(Thr422Ala), and he was kept on regular biotin and thiamine supplementation. Most recent follow up of brain MRI at age of 8-year showed regression course with reduction of lentiform and caudate nuclei size bilaterally associated with abnormal signals representing old insults. He is the 7^th^ and younger child of a consanguineous carrier parents with 5 carrier siblings and one normal sister, otherwise his family history was unremarkable.

**Case 15.** This is an 8-year-old Jordanian female, diagnosed with BTBGD when she was 2-year-6-month-old. She was born full-term via SVD with birth weight of 3 kg. She was apparently normal for one month and on breast feeding, until she was presented with irritability and abnormal movements, progressed to lethargy, and was followed by two episodes of generalized tonic clonic convulsions that were controlled with intravenous phenobarbitone. Brain CT showed multiple hyperdense areas in subthalamic region suggestive of metabolic disorder and EEG showed disturbed background activity reflecting transitional neonatal to infantile pattern with abnormal features that can be fit into metabolic disease. Few days later, she developed hypothermia, nystagmus, multiple episodes of apnea, desaturation, and respiratory acidosis, thus was shifted to PICU. Septic work up was normal except for mild elevated lactate level in blood and CSF. Echocardiogram showed patent foramen ovule with tiny closing patent ductus arteriosus, head ultrasound was normal and brain MRI revealed bilateral symmetric hyperintense signals in the midbrain / cerebral peduncles, as well as the basal ganglia and medial thalami There are multiple cortical/ subcortical and bilateral sub-insular T2 hyperintensities. Her throat swab tested positive to methicillin-resistant staphylococcus aureus (MRSA), and she was treated with clindamycin. At age of two-month, repeated brain MRI showed bilateral infarction with symmetrical restricted diffusion with reduced severity compared to previous MRI, suggestive of mitochondrial encephalomyopathy lactic acidosis and stroke-like episodes (MELAS), cerebral autosomal dominant arteriopathy with sub-cortical infarcts and leukoencephalopathy (CADASIL), Krabbe disease or cerebral infarctions, specifically Citrobacter, Klebsiella or Listeria. She also was noticed to have four limbs spasticity and lower limbs contractures along with brisk reflexes and clonus. The patient was treated with cefotaxime (Claforan), Ampicillin, metronidazole (Flagyl), acyclovir (Zovirax), ranitidine (Zantac), thiamine, vitamin B6, vitamin B complex, coenzyme Q10, L-Carnitine and ketogenic diet. After that her symptoms have improved and she was able to breath spontaneously, so she was discharged with multidisciplinary team follow ups. Molecular analysis at age of two-year-6-month confirmed the diagnosis of BTBGD with novel homozygous *SLC19A3* variant c.175T>C p.(Trp59Arg), so she was kept on biotin and thiamine supplementation. At age of 3-year, she was noted to have progressive upper limbs hypotonia more towards the distal extremities, despite receiving her medications due to delayed initiation of treatment. This case is a product of consanguineous marriage with two healthy siblings and a family history of infants and neonatal deaths in cousins. She is non-ambulatory and bedridden with microcephaly but no dysmorphic features, in addition to severe progressive GDD since birth as she never set, walk, or achieve speech. She is mechanical ventilator dependent and nasogastric tube feeding dependent for the past 10 months since the age of 7 years.

**Case 18.** This is a 4-year-old Kuwaiti male, who was born to carrier parents after full-term pregnancy via SVD with birth weight of 3 kg. He was completely healthy with normal developmental milestones. Past medical history revealed tooth extraction due to dental caries. At age of 2-year-11-month, he got COVID19 infection with low grade fever proceeded by unsteady gait and inability to move limbs. Brain MRI revealed bilateral symmetrical central necrosis of basal ganglia. Molecular diagnosis revealed a homozygous pathogenic *SLC19A3* variant c.1264A>G p.(Thr422Ala). He was started on biotin and thiamine supplementation.

**Case 19*.*** A 3-year-6-month-old Kuwaiti male was diagnosed with BTBGD at age of 3-year. He was born full-term (39-week gestation), via induced vaginal delivery due to oligohydramnios with birth weight of 2.7 kg. His neonatal history is unremarkable. Other than the occasional strabismus that was started at age one-year, he was completely healthy with normal developmental milestones. He had no dysmorphic features but noticed to have long eyelashes. The patient was vaccinated up to his age, except for the BCG vaccine which was missed. At age of 2-year, he presented with 10-day history of recurrent dystonic movements of the limbs, unsteady gait, abnormal gaze, on-off mouth deviation, slurred speech and pain while walking and limping, associated with irritability and fatigue last few days. His symptoms were triggered by profuse physical activity after walking in Mekka, Saudi Arabia. Brain CT showed multiple bilateral cerebral patchy hypodensities in frontal and parietal regions, cortical and subcortical in location, affecting the underlying cortical sulci as well as both basal ganglia regions. Brain MRI revealed multiple scattered hyperintense lesions at the cortical and subcortical cerebral parenchyma, as well as bilateral caudate , putamen and medial thalamic nuclei. Some of these areas showed diffusion restriction on DWI. EEG showed normal electrical activity. His symptoms resolved gradually within 10 days after the initiation of high doses of biotin and thiamine supplementation. Molecular genetic testing revealed a homozygous pathogenic variant c.1264A>G p.(Thr422Ala) in gene *SLC19A3* which confirm the diagnosis of BTBGD. He is the only child of consanguineous carrier parents. His mother has hypothyroidism and a history of three early miscarriages after the delivery of this child. His maternal aunt was diagnosed with absence seizure in childhood controlled with Depakene.
