# Supplementary material for: Biotin-thiamine responsive basal ganglia disease: a retrospective review of the clinical, radiological and molecular findings of cases in Kuwait with novel variants
Source: Orphanet J Rare Dis. 2023 Sep 5;18:271. doi: 10.1186/s13023-023-02888-y (PMC10478457; doi:10.1186/s13023-023-02888-y)
Supplement: Supplementary file 2 — Additional file 2: Table S1. Overview of individuals diagnosed with Biotin Thiamine Responsive Basal Ganglia Disease in Kuwait (n=21). Abbreviations: ASD, atrial septal defect; BG, basal ganglia; Bwt, birth weight; CADASIL, cerebral autosomal dominant arteriopathy with sub-cortical infarcts and leukoencephalopathy; CS, cesarian section; CSF, cerebral spinal fluid; CT, computed tomography; DWI, diffusion-weighted images; FT, full-term; f/u, follow up; FLAIR, fluid-attenuated inversion recovery; GDD, global developmental delay; HTN, hypertension; ID, intellectual disability; IDA, iron deficiency anemia; IEM, inborn errors of metabolism; Kg, kilogram; LBW, low birth weight; MELAS, mitochondrial encephalomyopathy lactic acidosis and stroke-like episodes; MRI, magnetic resonance imaging; MRSA, methicillin-resistant Staphylococcus aureus; MV, mechanical ventilator; NA, not applicable; NGT, nasogastric tube; NICU, neonatal intensive care unit; NNJ, neonatal jaundice; NVD, normal vaginal delivery, PDA, patent ductus arteriosus; PFO, patent foramen ovale; PICU, pediatric intensive care unit; PO, product of; RD, respiratory distress. *The reference transcript is NM_025243.4. [file 13023_2023_2888_MOESM2_ESM.docx]

|  | Case 1 | Case 2 | Case 3 | Case 4 | Case 5 | Case 6 | Case 7 | Case 8 | Case 9 | Case 10 | Case 11 | Case 12 | Case 13 | Case 14 | Case 15 | Case 16 | Case 17 | Case 18 | Case 19 | Case 10 | Case 21 |
| --- | --- | --- | --- | --- | --- | --- | --- | --- | --- | --- | --- | --- | --- | --- | --- | --- | --- | --- | --- | --- | --- |
| *SLC19A3* DNA Variant^a^ | c.1264A>G (homozygous) | c.1264A>G (homozygous) | c.1264A>G (homozygous) | c.1264A>G (homozygous) | c.1264A>G (homozygous) | c.1264A>G (homozygous) | c.1264A>G (homozygous) | c.1264A>G (homozygous) | c.1264A>G (homozygous) | c.1264A>G (homozygous) | c.1264A>G (homozygous) | c.1264A>G (homozygous) | c.1264A>G (homozygous) | c.1264A>G (homozygous) | c.1264A>G (homozygous) | c.1264A>G (homozygous) | c.1264A>G (homozygous) | c.1264A>G (homozygous) | c.1264A>G (homozygous) | c.1264A>G (homozygous) | c.1264A>G (homozygous) |
| *SLC19A3* Protein Variant | p.Thr422Ala | p.Thr422Ala | p.Thr422Ala | p.Thr422Ala | p.Thr422Ala | p.Thr422Ala | p.Thr422Ala | p.Thr422Ala | p.Ala318Thr | p.Thr422Ala | p.Thr422Ala | p.Thr422Ala | p.Thr422Ala | p.Thr422Ala | p.Trp59Arg | p.Thr422Ala | p.Thr422Ala | p.Thr422Ala | p.Thr422Ala | p.Thr422Ala | p.Thr422Ala |
| Novel Variant | No | No | No | No | No | No | No | No | Yes | No | No | No | No | No | Yes | No | No | No | No | No | No |
| Age at diagnosis | 32 years | 20 years | 2.5 years | 3 years | 5 years | 2 years | 7 years | 2 years 2 months | 3 years | 4.5 years | 2 years | 3 years | 1 year (by screening) | 3 years | 2.5 years | 2.5 years, (by screening) | 1.5 years | 2 years 11 months | 3 years | 2 years | At birth, by screening) |
| Current age | 36 years | 25 years | 23 years | 18 years | 17 years | 15.5 years | 15 years | 12 years | 10 years | 10 years | 9 years | 9 years | 8 years | 8 years | 8 years | 6 years | 5years | 4 years | 3 years | 2.5 years | 2 years |
| Gender | Male | Male | Female | Male | Male | Female | Female | Female | Female | Male | Female | Female | Male | Male | Female | Male | Female | Male | Male | Female | Female |
| Nationality | Kuwaiti | Non-Kuwaiti | Kuwaiti | Kuwaiti | Kuwaiti | Kuwaiti | Kuwaiti | Kuwaiti | Kuwaiti | Kuwaiti | Kuwaiti | Kuwaiti | Kuwaiti | Kuwaiti | Jordanian | Kuwaiti | Kuwaiti | Kuwaiti | Kuwaiti | Kuwaiti | Kuwaiti |
| Consanguinity | Yes | Yes | Yes | Yes | Yes | Yes | Yes | Yes | Yes | Yes | Yes | No | Yes | Yes | Yes | Yes | Yes | Yes | Yes | Yes | Yes |
| Family history | -2 affected cousins **(Case 2 &17)**  -Leigh disease  -Type 1 diabetes mellitus  -Mitochondrial dysfunction | -2 affected cousins **(case 1 & 17)**  -Cousin with Leigh disease **(Case 1)** | -Affected sibling **(Case 4)** and niece **(Case 20)** | -Affected sibling **(Case 3)** and niece **(Case 20)** | -Unremarkable | - Affected sibling **(Case 8)**  - 2 paternal cousins with Leigh’s disease | - Suspected elder sibling with clinical and radiological findings of BTBGD but not confirmed genetically | - Affected sibling **(Case 6)** | -Unremarkable | -Affected sibling **(Case 16)** | -2 affected siblings **(Case 13&21)** | -A relative with metabolic disease | -2 affected siblings **(Case 11&21)** | -Unremarkable | -Infant deaths in cousins | -Affected sibling **(Case 10)** | - 2 affected cousins **(case 1&2)** | -Unremarkable | -3 early miscarriages  -Hypothyroidism  -Absence seizure on Depakene | -Affected maternal uncle & aunt **(Case 3&4)** | -2 affected siblings **(Case 11&13)** |
| GDD/ID | + | - | + | + | + | - | - | - | - | - | - | - | - | - | + | - | - | - | - | - | - |
| Dysmorphic features | Hypertelorism, telecanthus, bilateral epicanthic fold synophy, prominent nasal bridge, anteverted nostril, high arched palate, interdigital webbing, long fingers, wide space between the first and second toes and shawl scrotum | - | - | - | - | - | - | - | - | - | - | - | - | - | - | - | - | - | Long eyelashes | - | - |
| Skeletal findings | - | - | Scoliosis | - | Scoliosis | - | Scoliosis | - | - | - | - | - | - | - | - | - | - | - | - | - | - |
| Drowsiness/ irritability/ lethargy | + | + | + | - | + | - | - | - | - | + | + | + | - | + | + | - | - | - | + | - | - |
| Convulsions | + | + | - | + | + | + | + | - | + | - | - | - | - | - | + | - | - | - | - | - | - |
| Hypertonia / contractures Dystonia / Choreoathetosis | - | + | + | + | + | - | + | - | + | - | + | - | - | + | + | - | - | - | + | + | - |
| Tremor | - | + | - | - | - | - | - | - | - | + | - | - | - | + | - | - | + | - | - | - | - |
| Hypotonia | + | + | - | + | - | - | - | - | - | - | + | - | - | + | + | - | - | - | - | - | - |
| Poor head control | - | + | - | - | + | - | - | - | - | - | - | - | - | - | - | - | - | - | - | - | - |
| Limping/unsteady/ataxic gait | - | + | + | + | - | + | + | + | + | + | + | + | - | + | - | - | - | + | + | + | - |
| Dysarthria/ drooling/ nasal speech | - | + | + | + | - | - | + | - | + | - | + | - | - | - | - | - | + | - | - | - | - |
| Nystagmus / strabismus / abnormal gaze | - | - | + | - | - | + | - | - | - | - | - | - | - | - | + | - | - | - | + | + | - |
| Drooling | - | + | - | - | + | - | - | - | + | - | - | - | - | - | - | - | - | - |  | + | - |
| Chronic constipation | - | - | - | - | + | - | - | - | - | - | - | - | - | - | - | - | - | - | - | - | - |
| PICU admission | - | - | - | - | - | - | - | - | - | - | - | - | - | +,  For irritability and acute encephalopathy | +,  For apnea, desaturation and respiratory acidosis and MRSA in throat swab | - | - | - | - | - | - |
| Neonatal history | FT, NVD | FT, CS, Bwt 2.5kg | Not reported | FT, NVD, Bwt 2.5 kg | FT, NVD, Bwt 3.5kg | FT, NVD, Bwt 3 kg | FT, NVD | FT, NVD, Bwt 3.5 kg | FT, NVD | PO 28, NVD, Bwt 800kg | FT, NVD, Bwt 3.5kg | FT, NVD, Bwt 3.1kg | FT, NVD, Bwt >4kg | FT, NVD, Bwt 2.9kg | FT, NVD, Bwt 3kg | FT, NVD, Bwt 1kg | FT, NVD, Bwt 3kg | FT,NVD, Bwt 3kg | FT, NVD, Bwt 2.7kg | FT, NVD | FT, NVD, Bwt 3.5 kg |
| Perinatal history | Gestational HTN on treatment & maternal IDA on iron injections, Child NNJ & RD | Gestational HTN on methyldopa | Not reported | - | - | - | - | - | - | NICU admission for LBW | - | - | - | - | PFO, tiny closing PDA | ASD | - | - | Maternal hypothyroidism & 3 early miscarries after this child  Maternal aunt with childhood absence seizure controlled with Depakene | - | - |
| Other | - | - | - | - | - | - | - | - | - | - | - | - | - | - | Mild elevated lactate level in blood and CSF | - | - | - | - | - | - |
| Brain MRI | Bilateral signal alteration in lentiform nucleus; f/u: subacute necrotizing encephalopathy | Bilateral corpus striatum signal alteration with lentiform dystrophic calcification associated with normal brain parenchyma; f/u: reduction in size with some atrophic changes | Bilateral corpus striatum signal alteration with central necrosis at the head of caudate nuclei, restricted diffusion at the outer aspect of lentiform nuclei and central dysmorphic calcifications | Bilateral caudate, putamen and external capsule atrophy sparing globes pallidus and sub-insular regions, with multiple T2 hyperintense cystic foci of necrosis | Bilateral symmetrical involvement of the basal ganglia and thalamus. | Acute diffuse gray matter metabolic disease involving deep gray matter and basal ganglia suggestive of mitochondrial disease | Multiple bright hyperintense lesions again in the basal ganglia and thalami with conspicuous subcortical involvement. | Initially normal, then at age of 6 years it progressed to develop bilateral putamen mild cystic changes | Cortical and subcortical hyperintensities at both cerebral hemispheres and subtle cerebellar changes, as well as caudate and putamen bilaterally. | Symmetrical bilateral basal ganglia involvement affecting the lentiform nuclei as well as the medial portion of the thalamic nuclei | Bilateral symmetrical hyperintensity with diffused restriction and swelling in caudate nuclei and putamen, sparing the globus pallidus | Bilateral symmetrical central necrosis of basal ganglia | Not done | Symmetrical diffusely swollen lentiform and caudate nuclei with lentiform fork sign; f/u: showed regression course with reduction of lentiform and caudate nuclei size bilaterally associated with abnormal signals representing old insults | Bilateral symmetric hyperintense signals in the midbrain / cerebral peduncles, as well as the basal ganglia and medial thalami There are multiple cortical/ subcortical and bilateral sub-insular T2 hyperintensities.. | Not Done | Bilateral symmetrical central necrosis of basal ganglia | Bilateral symmetrical central necrosis of basal ganglia | Multiple scattered hyperintense lesions at the cortical and subcortical cerebral parenchyma, as well as bilateral caudate , putamen and medial thalamic nuclei. (l) Some of these areas showed diffusion restriction on DWI. | Hyperintensity of bilateral putamen, representing atrophy with central necrosis. | Not done |
| Current medications | Valproic acid (Depakene), vitamin B6, thiamine, clonazepam drops (Rivotril), biotin supplement, coenzyme Q10 & carnitine | Vitamin B6, vitamin B complex, L-carnitine syrup, coenzyme Q10, biotin and thiamine supplementation | Biotin and thiamine supplementation | Vitamin B6, vitamin B complex, biotin and thiamine supplementation | Carbamazepine (Tegretol), clonazepam and baclofen, biotin and thiamine supplementation | Coenzyme Q10, L.carnitine, vitamin B6, thiamine, and B complex supplementation | Biotin, thiamine, Vitamin B6, Keppra  (Non-compliance with improper dosing by caregiver) | Coenzyme Q10, L.carnitine, vitamin B6, thiamine, and B complex supplementation | Biotin and thiamine supplementation  ( Non-compliance & disadvantage home situation) | Biotin and thiamine supplementation | Biotin and thiamine supplementation | Biotin and thiamine supplementation | Biotin and thiamine supplementation | Biotin and thiamine supplementation | Biotin, thiamine, vitamin B6, vitamin B complex, coenzyme Q10, L-carnitine and ketogenic diet. | Biotin and thiamine supplementation | Biotin and thiamine supplementation | Biotin and thiamine supplementation | Biotin and thiamine supplementation | Biotin and thiamine supplementation | Biotin and thiamine supplementation |
| Residual neurological deficit | + | + | - | - | +  wheelchair bound | - | + | - | -  but multiple relapses and admissions due to stopping medication by caregiver | - | - | - | - | + | +  MV dependent,  NGT feeding,  Bed-ridden | - | - | - | - | - | - |

**Supplementary Table S3**: Overview of individuals diagnosed with Biotin Thiamine Responsive Basal Ganglia Disease in Kuwait (n=21).

**Abbreviations**: ASD, atrial septal defect; BG, basal ganglia; Bwt, birth weight; CADASIL, cerebral autosomal dominant arteriopathy with sub-cortical infarcts and leukoencephalopathy ;CS, cesarian section; CSF, cerebral spinal fluid; CT, computed tomography; DWI, diffusion-weighted images; FT, full-term; f/u, follow up; FLAIR, fluid-attenuated inversion recovery; GDD, global developmental delay; HTN, hypertension; ID, intellectual disability; IDA, iron deficiency anemia; IEM, inborn errors of metabolism; Kg, kilogram; LBW, low birth weight; MELAS, mitochondrial encephalomyopathy lactic acidosis and stroke-like episodes; MRI, magnetic resonance imaging; MRSA, methicillin-resistant Staphylococcus aureus; MV, mechanical ventilator; NA, not applicable; NGT, nasogastric tube; NICU, neonatal intensive care unit; NNJ, neonatal jaundice; NVD, normal vaginal delivery, PDA, patent ductus arteriosus; PFO, patent foramen ovale; PICU, pediatric intensive care unit; PO, product of; RD, respiratory distress;

***The reference transcript is NM_025243.4**
