# Supplementary material for: Biotin-thiamine responsive basal ganglia disease: a retrospective review of the clinical, radiological and molecular findings of cases in Kuwait with novel variants
Source: Orphanet J Rare Dis. 2023 Sep 5;18:271. doi: 10.1186/s13023-023-02888-y (PMC10478457; doi:10.1186/s13023-023-02888-y)
Supplement: Supplementary file 3 — Additional file 3: Fig. S1. Age of presentation, age of diagnosis and current age of individuals diagnosed with Biotin Thiamine Responsive Basal Ganglia Disease in Kuwait (n=21). [file 13023_2023_2888_MOESM3_ESM.pptx]

## Slide 1
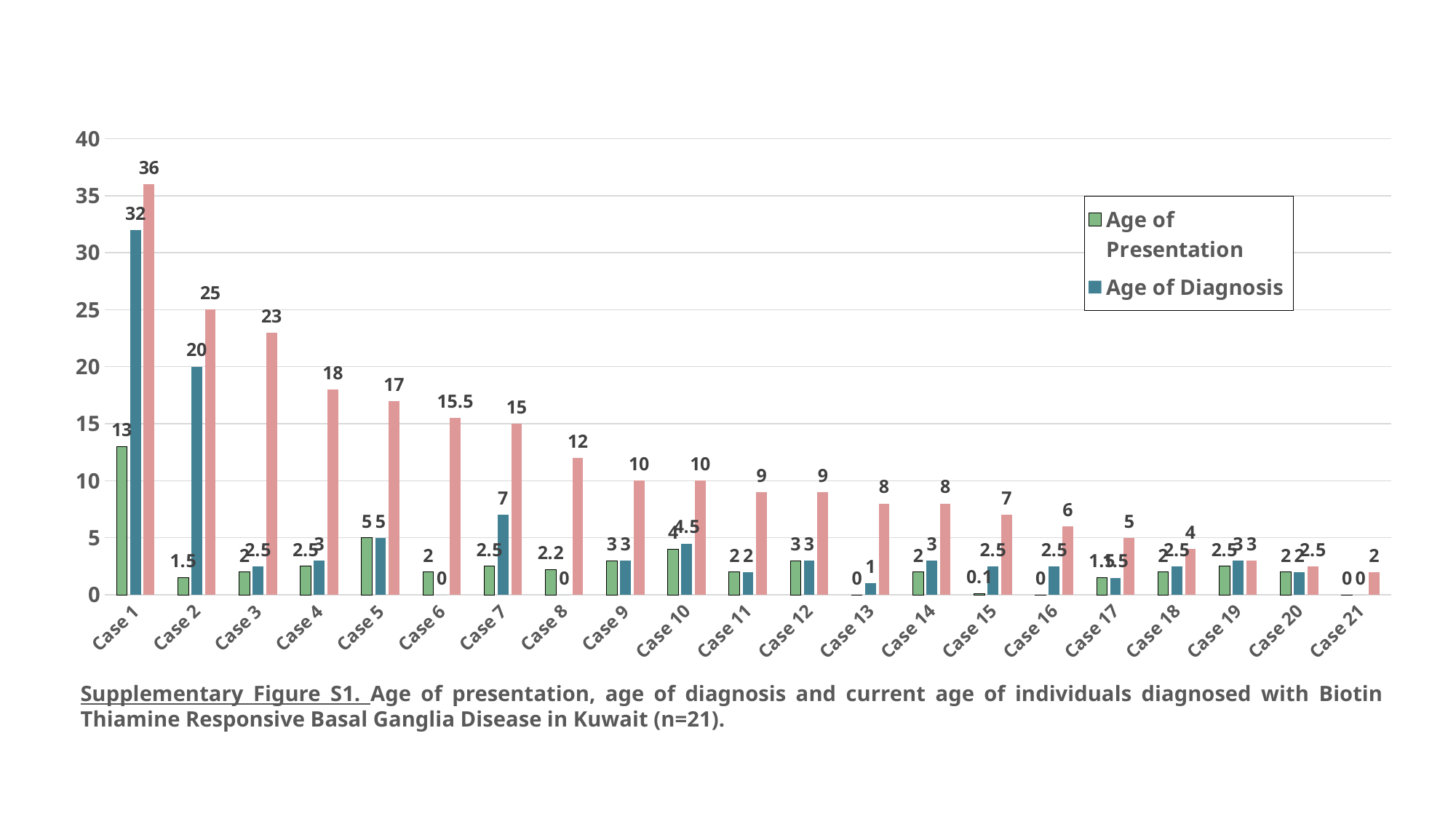

### Chart
| Category | Age of Presentation | Age of Diagnosis | Current Age |
|---|---|---|---|
| Case 1 | 13.0 | 32.0 | 36.0 |
| Case 2 | 1.5 | 20.0 | 25.0 |
| Case 3 | 2.0 | 2.5 | 23.0 |
| Case 4 | 2.5 | 3.0 | 18.0 |
| Case 5 | 5.0 | 5.0 | 17.0 |
| Case 6 | 2.0 | 0.0 | 15.5 |
| Case 7 | 2.5 | 7.0 | 15.0 |
| Case 8 | 2.2 | 0.0 | 12.0 |
| Case 9 | 3.0 | 3.0 | 10.0 |
| Case 10 | 4.0 | 4.5 | 10.0 |
| Case 11 | 2.0 | 2.0 | 9.0 |
| Case 12 | 3.0 | 3.0 | 9.0 |
| Case 13 | 0.0 | 1.0 | 8.0 |
| Case 14 | 2.0 | 3.0 | 8.0 |
| Case 15 | 0.1 | 2.5 | 7.0 |
| Case 16 | 0.0 | 2.5 | 6.0 |
| Case 17 | 1.5 | 1.5 | 5.0 |
| Case 18 | 2.0 | 2.5 | 4.0 |
| Case 19 | 2.5 | 3.0 | 3.0 |
| Case 20 | 2.0 | 2.0 | 2.5 |
| Case 21 | 0.0 | 0.0 | 2.0 |Supplementary Figure S1. Age of presentation, age of diagnosis and current age of individuals diagnosed with Biotin Thiamine Responsive Basal Ganglia Disease in Kuwait (n=21).
